# Supplementary figures and images for: SARS-CoV-2 neutralizing antibodies: Longevity, breadth, and evasion by emerging viral variants
Source: PLoS Med. 2021 Jul 6;18(7):e1003656. doi: 10.1371/journal.pmed.1003656 (PMC8291755; doi:10.1371/journal.pmed.1003656)

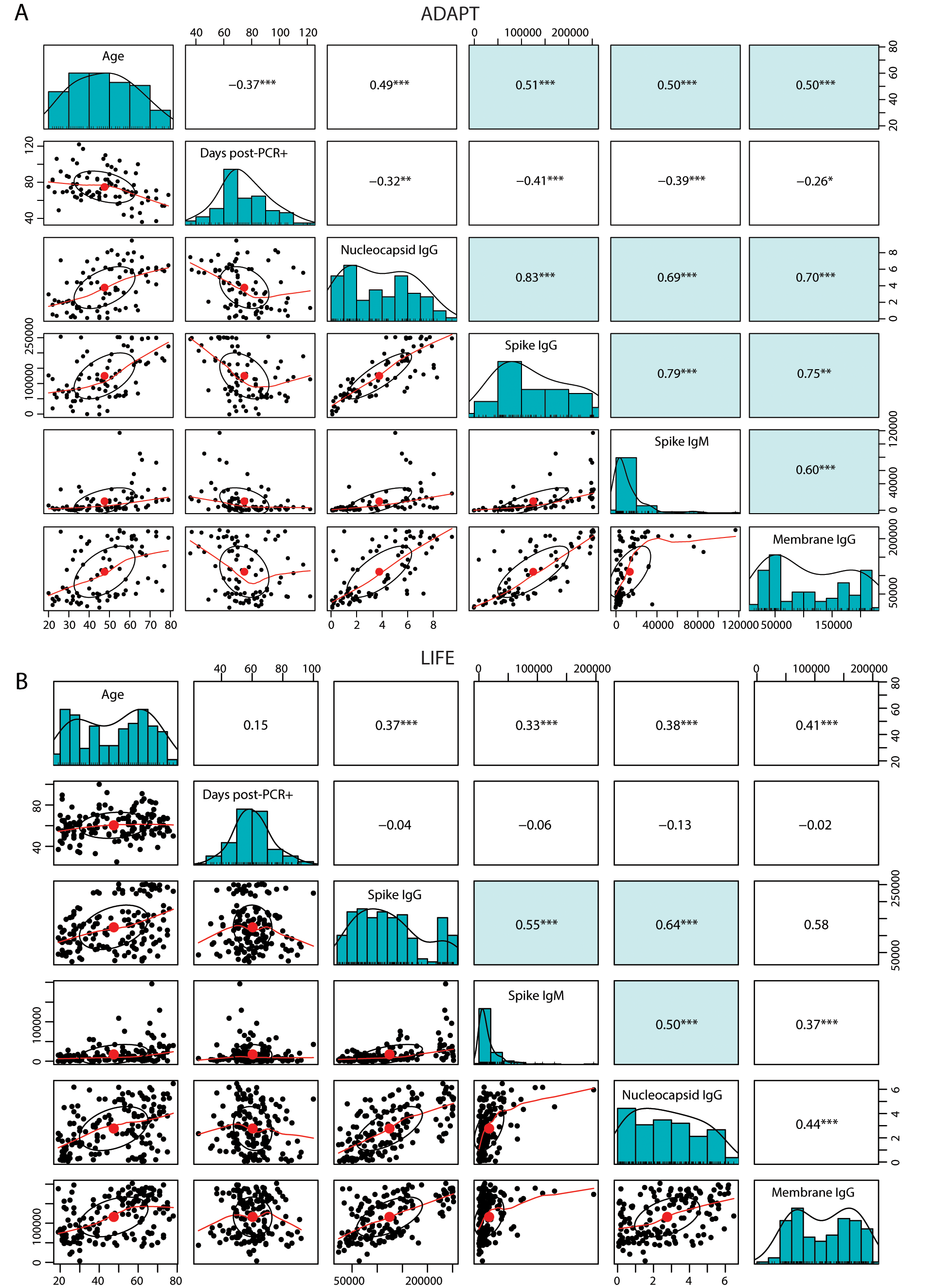

Supplement: S1 Fig — Correlation matrices for antibody responses and demographic data in ADAPT (A) and LIFE (B). Continuous measures of demographics (age, days post-PCR positivity) and antibody titers (Nucleocapsid, Spike IgG and IgM, and Membrane), were compared. R-square values are placed in the boxes (top-right) with P value significance shown in *. Bottom-left show correlation plots with Loess line (red). PCR, polymerase chain reaction. (TIF) [file pmed.1003656.s001.tif]

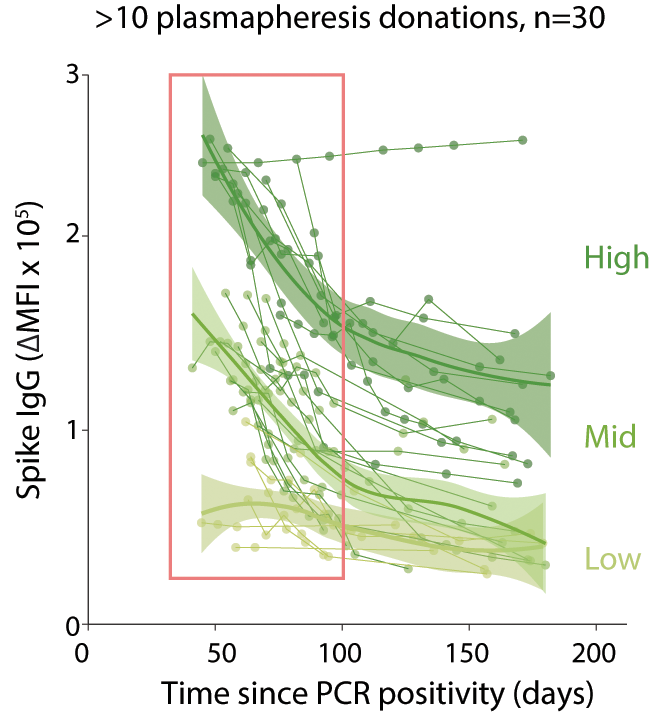

Supplement: S2 Fig — Spike IgG titer over time is shown from n = 30 LIFE donors who underwent >10 plasmapheresis donations. Despite high donations, donors with high Spike IgG titers decreased but stabilized at mid-levels, whereas donors with mid to low Spike IgG titers stabilized at low titers. No donors seroreverted nor became negative for Spike IgG. (TIF) [file pmed.1003656.s002.tif]

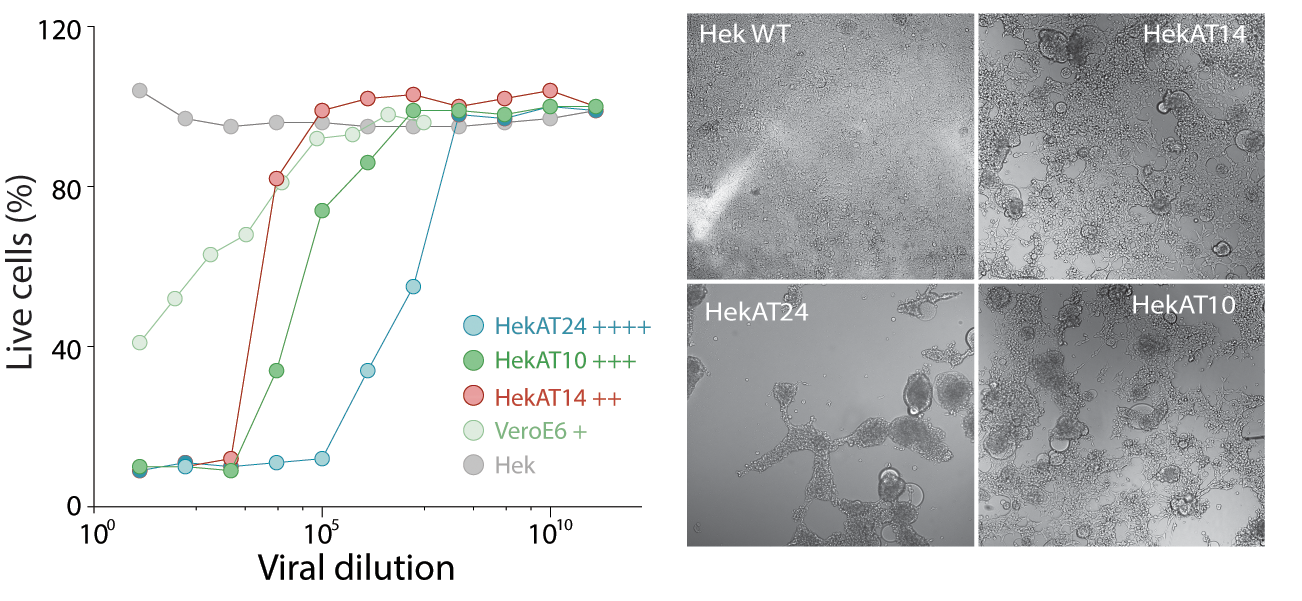

Supplement: S3 Fig — HekAT clonal cell lines and VeroE6 were infected with serially diluted SARS-CoV-2 and monitored for CPE at 72 hours postinfection. (A) Cell nuclei were stained with NucBlue and CPE quantified as % live cells. HekAT clones showed varying degrees of permissiveness to SARS-CoV-2 infection with HekAT24 being orders of magnitude more susceptible than VeroE6. WT Hek293T cells were refractory to infection and were used as negative control. (B) Bright-field images showing CPE in WT Hek and HekAT clonal lines. Images were acquired using InCell high throughput imaging system. Magnification is 10× for all images. Representative images are shown. CPE, cytopathic effect; SARS-CoV-2, Severe Acute Respiratory Syndrome Coronavirus 2; WT, wild-type. (TIF) [file pmed.1003656.s003.tif]

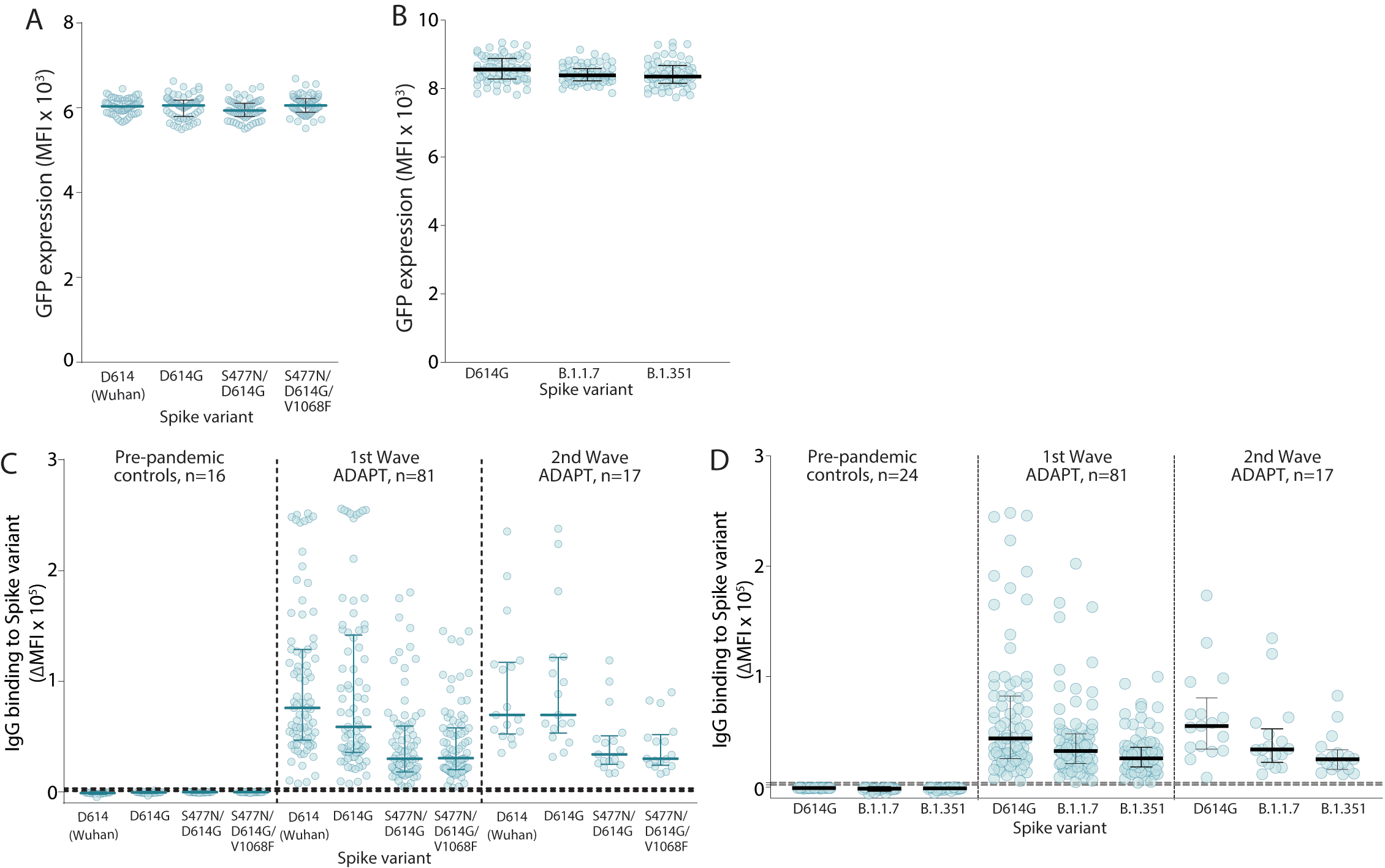

Supplement: S4 Fig — (A and B) Expression of GFP reporter molecule expressed via the transcription of Spike-GFP monocistron in the pIRES2 plasmids was similar across all analyzed variants, allowing accurate comparisons between IgG levels between variants. (B and C) Pre-pandemic controls were below the threshold, whereas all first and second wave of ADAPT samples were above the positive threshold (Control + 4SD) in (B) all D614G Spike variants and in (C) VOC B.1.1.7 (United Kingdom) and B.1.351 (South African). VOC, variant of concern. (TIF) [file pmed.1003656.s004.tif]

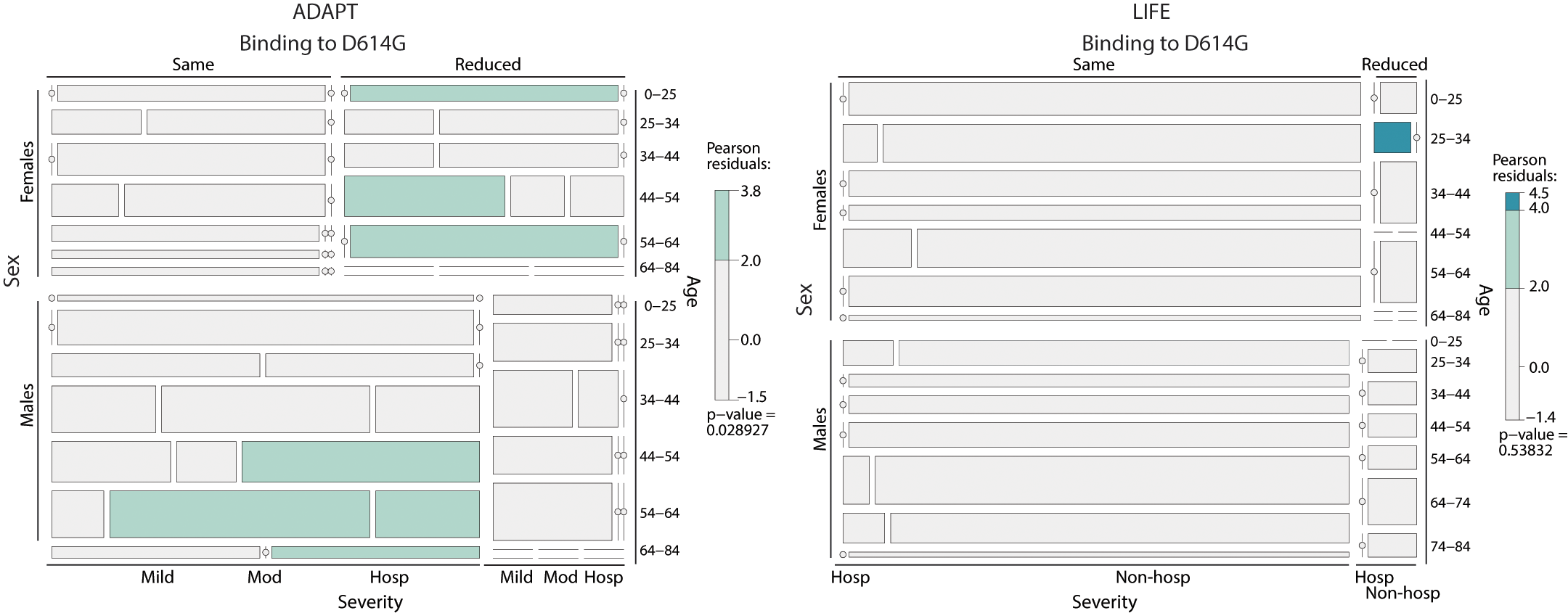

Supplement: S5 Fig — Mosaic plots of ADAPT (left) and LIFE (right) show individuals with restricted binding to Spike variant, i.e., reduced binding to D614G, were more likely to be females. (TIF) [file pmed.1003656.s005.tif]

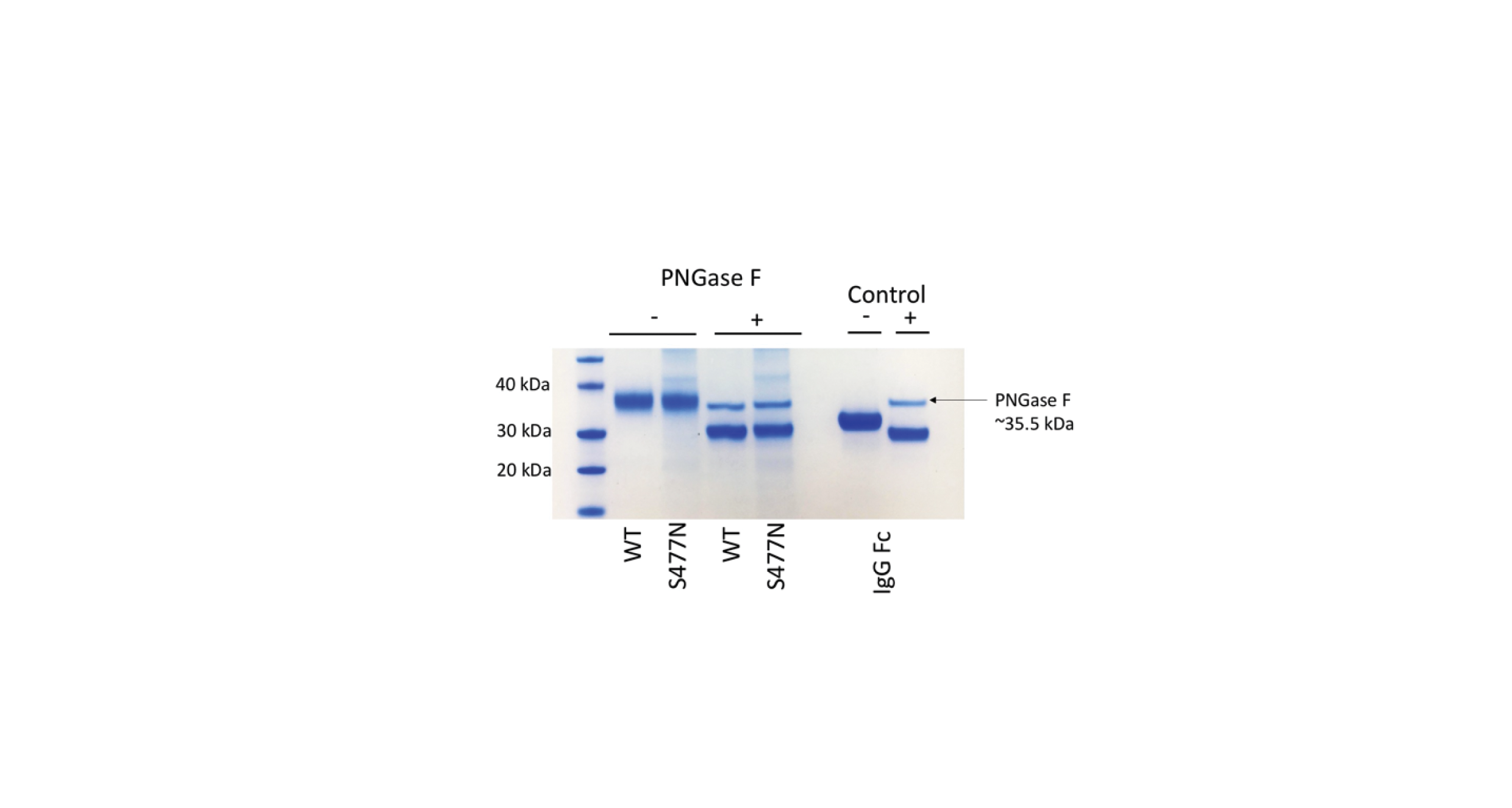

Supplement: S6 Fig — SDS-PAGE of purified Wuhan-1 (WT) and S477N RBD expressed in human Expi293 cells under reducing conditions (stained with Coomassie blue). N-linked carbohydrates were removed by treatment with PNGaseF (+). No additional N glycosylation could be observed in S477N compared to Wuhan-1 (WT). The Fc region of human IgG1 (carrying a single glycosylation site at position 297) was used as a control. WT, wild-type. (TIF) [file pmed.1003656.s006.tif]

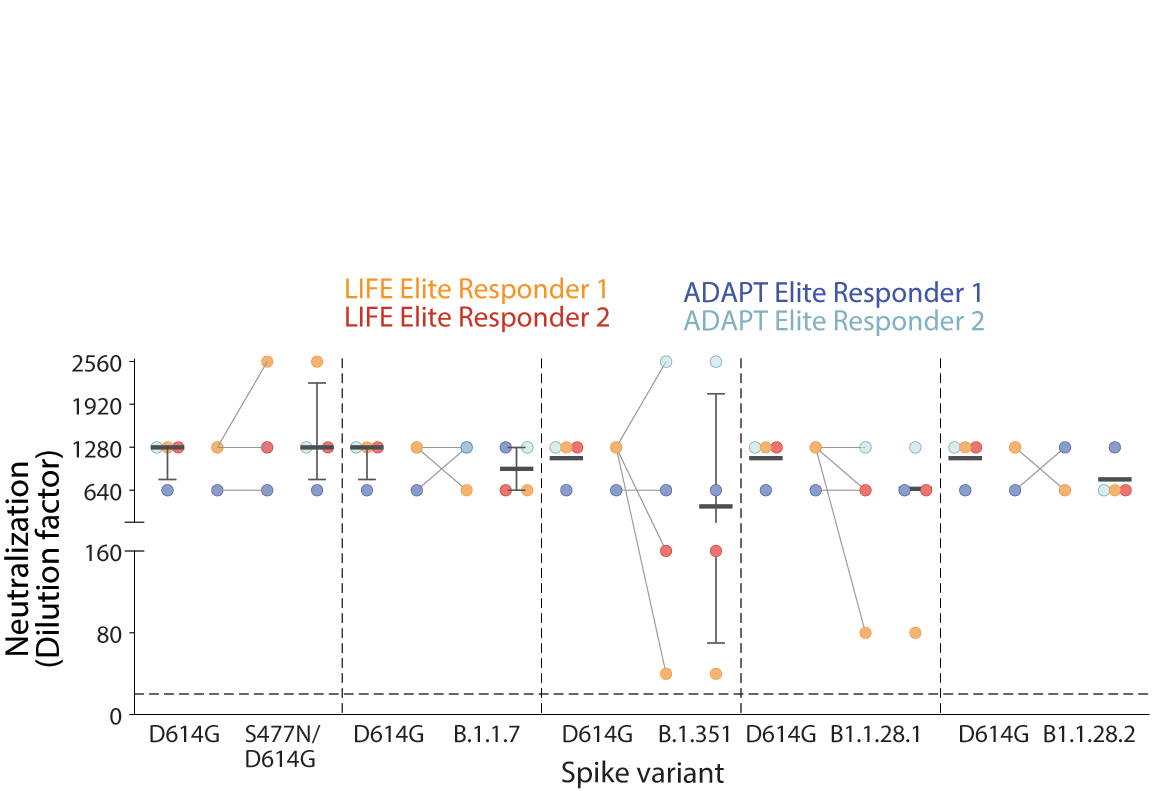

Supplement: S7 Fig — (I) High and elite sera maintained high titers of neutralization to live VOC B.1.1.7, B1.351, B1.1.28.1, and B1.1.28.2. SARS-CoV-2, Severe Acute Respiratory Syndrome Coronavirus 2; VOC, variant of concern. (TIF) [file pmed.1003656.s007.tif]

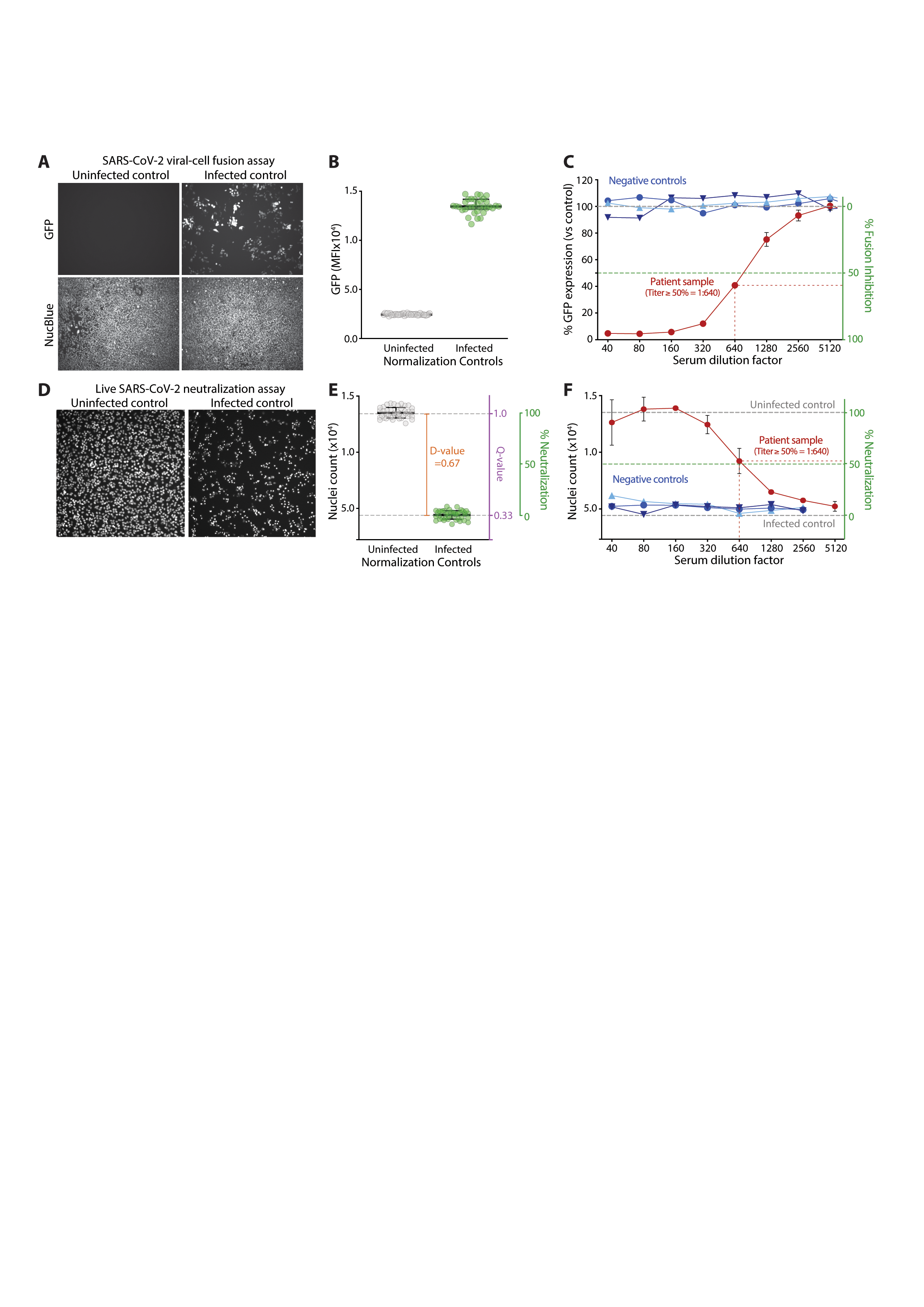

Supplement: S8 Fig — (A) Uninfected and infected ACE2-Hek 293T cells in Spike-driven virus–cell fusion assay. Spike-mediated entry of lentiviral/pseudoviral particles resulted in robust GFP expression in infected cells, while the uninfected cells showed no GFP signal (top). Cell nuclei were stained with NucBlue (bottom). (B) Mean intensity values of GFP signal in uninfected and infected control cells. Data points represent pooled technical replicates. (C) Serum titration curves for a high neutralizing convalescent serum and sera from pre-pandemic healthy controls. Dilution resulting in 50% reduction in GFP expression relative to the infected control was taken as the cutoff to determine fusion inhibition titers. (D) Uninfected and infected VeroE6 cells in SARS-CoV-2 live neutralization assay. Cell nuclei were stained with NucBlue. Infection with live virus resulted in abundant cytopathic effect and cell death at 72 hours postinfection, leading to lower cell numbers compared to uninfected control. (E) The average uninfected nuclei counts (data points represented pooled technical replicates) was defined as 100% neutralization, whereas the average of infected nuclei counts was defined as 0% neutralization. The % viral neutralization of a sample was calculated using the formula described in Materials and methods. (F) Serum titration curves for a high neutralizing convalescent serum and sera from pre-pandemic healthy controls. The cutoff for determining the neutralization titer of diluted serum samples was ≥50%. Magnification is 10× for all images. Representative data from 2 experiments are shown. Mean and standard deviation are shown. SARS-CoV-2, Severe Acute Respiratory Syndrome Coronavirus 2. (TIF) [file pmed.1003656.s008.tif]
