# Supplementary material for: SARS-CoV-2 neutralizing antibodies: Longevity, breadth, and evasion by emerging viral variants
Source: PLoS Med. 2021 Jul 6;18(7):e1003656. doi: 10.1371/journal.pmed.1003656 (PMC8291755; doi:10.1371/journal.pmed.1003656)
Supplement: S1 Table — (PDF) [file pmed.1003656.s009.pdf]

S1 Table. Nomenclature of SARS-CoV-2 and emerging variants.

**Virus**	**Other names***	**Country first detected**	**WHO Nomenclature**	**GISAID code**
D614	19A	China	N/A	
D614G	B.1.319/19B	China	N/A	NSW2713
D614G/S477N	20F	Australia	N/A	NSW712
B.1.1.7	20I	United Kingdom	Alpha	NSW3878
B.1.351	20H	South Africa	Beta	NSW3872
B.1.1.28.1	P1/20J	Brazil	Gamma	NSW4318
B.1.1.28.2	P2	Brazil	Zeta	NSW-R0099

* Pango lineage and clades

GISAID, Global Initiative on Sharing Avian Influenza Data; SARS-CoV-2: Severe Acute Respiratory Syndrome Coronavirus 2; WHO, World Health Organization.
