# Supplementary material for: SARS-CoV-2 neutralizing antibodies: Longevity, breadth, and evasion by emerging viral variants
Source: PLoS Med. 2021 Jul 6;18(7):e1003656. doi: 10.1371/journal.pmed.1003656 (PMC8291755; doi:10.1371/journal.pmed.1003656)
Supplement: Attachment — Submitted filename: Review POLSMed 02232021.docx [file pmed.1003656.s010.docx]

In this article, Tea et al. analyze the magnitude, functionality and breadth of the humoral response against SARS-Cov2 in two cohorts of individuals that had passed COVID-19. The authors propose a methodology based on flow cytometry and fluorescence imagen analysis as an alternative to more conventional ELISA/Luminescence techniques. The study analyzes the longevity of the SARS-Cov2 specific IgG and IgM for seven months after remission. In agreement with previous reports, they find an initial drop in the serum concentrations of IgG and IgM during the first three months after remission with a stabilization of the IgG levels at later times. They show that the responses have a wide breadth against different viral proteins targeting the membrane, envelope and nucleocapside proteins in addition to the spike protein.

They also confirmed previous reports that demonstrate a correlation between the magnitude and breadth of the response and the neutralizing activity. In addition, they characterize a subgroup of individuals with a superior neutralizing activity that correlates with high titters of IgM.

Finally, they show that the antibodies generated against SARS-CoV-2 during the first COVID-19 wave had reduced immunoreactivity and neutralization potency to emerging Spike variants.

The manuscript is well written, and the authors adequately address some discrepancies in their experimental setup that is the foundation of the most substantial parts of the study.

While the results confirm previous reports about the antibody kinetics and addresses important questions about the humoral responses against SARS-Cov2, the methodology as it is presently described raises some questions that, in my opinion should be addressed before publication:

Missing definition of neutralizing activity: fusion inhibition and neutralization are not defined anywhere in text. It is unclear if neutralization is defined as 100%, 80% or 50% of the maximal possible infection. Although in Figure 4 the authors show a neutralization titration, it lacks control experiments. The same happens with fusion inhibition assays: the authors speak of inhibition of the fusion between virus and cell membrane without specifying at what level or threshold the assay is defined as positive/negative.

Without proper referents showing fluorescence histograms and/or fluorescence dynamic range, it is exceedingly difficult to accurately comprehend the implications derived from the results shown in Figures 2 and 3.

As a minor point:

The authors make emphasis on the sensitivity and adequacy of the flow cytometry technique to assess quantification of immunoglobulins. The method is a cumbersome technique that requires transfection of HEK293 cells (it is unclear if is a stable or transitory transfection), cell permeabilization and staining of the cells suspension to be read in a flow cytometer. It is difficult to see how this method would be a viable tool to replace an indirect ELISA utilizing purified recombinant viral spike variants in terms of labor, cost, and sensitivity.

In summary: The study addresses important questions about the magnitude, longevity, and functionality of the humoral responses in COVID19 patients. It is well designed and properly discussed although the significance and scope of the results are not clear due to the lack of metrics in the fusion and neutralization assays. I think the work deserves serious consideration for publishing once these issues have been addressed.
