# Supplementary material for: SARS-CoV-2 neutralizing antibodies: Longevity, breadth, and evasion by emerging viral variants
Source: PLoS Med. 2021 Jul 6;18(7):e1003656. doi: 10.1371/journal.pmed.1003656 (PMC8291755; doi:10.1371/journal.pmed.1003656)
Supplement: Attachment — Submitted filename: FTea SARS-COV2_PlosMedicine response to reviewers_Rev.docx [file pmed.1003656.s011.docx]

**Point-by-point response to Reviewers:**

We would like to thank Reviewer 1, Reviewer 2, and Reviewer 3 for reviewing our manuscript. All amendments to the manuscript have been underlined (please note that the page number refers to the revised version of our manuscriptin the marked up manuscript file).

**Reviewer 1**

Thank you to Reviewer 1. No change was made.

**Reviewer 2**

1. **Missing definition of neutralizing activity: fusion inhibition and neutralization are not defined anywhere in text. It is unclear if neutralization is defined as 100%, 80% or 50% of the maximal possible infection**. **Although in Figure 4 the authors show a neutralization titration, it lacks control experiments. The same happens with fusion inhibition assays. The authors speak of inhibition of the fusion between virus and cell membrane without specifying at what level or threshold the assay is defined as positive/negative.Without proper referents showing fluorescence histograms and/or fluorescence dynamic range, it is exceedingly difficult to accurately comprehend the implications derived from the results shown in Figures 2 and 3**.

We thank Reviewer 2 for these comments. Fusion inhibition was defined as 50% reduction in GFP expression relative to the control group infected with the virus particles without any convalescent serum treatment, and the cut-off for determining the neutralization endpoint titre of diluted serum samples was set to ≥50% neutralization.

Adding to unifected and infected cells, both virus-cell fusion and neutralizatiuon assays (and Flow cytometry cell-based assay for detection of SARS-CoV-2 antibodies, please see below for Pt 1 Reviewer 3 were validated with a panel of 10 National Institute for Biological Standards and Control (NIBSC) standards. These NIBSC standards were distributed during the CS678 protocol for the World Health Organization (WHO) collaborative study to establish the first International Standard for anti-SARS-CoV-2 antibody and Reference Panel, which our laboratory participated in. We have added a supplementary figure and its legend (the new Supplementary Figure 8) illustrating the methods and controls used in the virus-cell fusion and the live virus neutralization assays.

We understand the necessity of setting up the threshold. In our experiments, we establish the cut-off of positivity for fusion inhibition and neutralization at 1:40. Accordingly, both fusion inhibition and neutralization of live virus was considered as absent when titers were below 1:40. This dilution was selected during validation experiments in which diagnostic sensitivity and specificity (>90% and >99%, respectively) were established using a panel of 167 convalescent RT-PCR-confirmed adult serum and 100 pre-pandemic negative serum samples collected prior to December 2019. Of note, these diagnostic parameters were comparable to those of other commercially available serology assays (Walker et, 2020). Results shown in Figures 2 and 3 were similar when only positive fusion inhibition and neutralization titers (1:40) were included.

We have now edited the manuscript:

- Page 10, line 215, we have now added: “These results were similar when only positive fusion and neutralization were included, i.e. titers above 1:40.”
- Page 18, line 415 (Discussion): we have now added “and validation controls”
- Page 24, line 570 (Materials and Methods): we have added: “Uninfected cells were run in parallel to account for background fluorescence signal. In infected cells, Spike driven entry of lentiviral particles resulted in strong GFP expression while the uninfected controls were negative for GFP (Fig. S8A, 8B).”
- Page 25, line 574 (Materials and Methods): we have added “Serum dilution resulting in 50% reduction in GFP expression relative to the infected control was used to determine virus-cell fusion inhibition titers (Fig. S8B, 8C). The cut-off of positivity for virus-cell fusion inhibition was 1:40. Accordingly, fusion inhibition was considered as absent when titers were below 1:40. Pre-pandemic healthy control sera (Privigen USA, P100103528, 2091200221, P100007516) showed no fusion inhibition and hence no reduction in GFP expression, thus confirmimg the specificity of the assay (Fig. S8C). Of the 10 NIBSC standards (CS678 WHO protocol), five were positive in the assay with mid and high virus-cell fusion inhibition, consistent with the confirmed results from the NIBSC.”
- Page 26, line 599 (Materials and Methods): we have added “(Fig. S8D, 8E). The cut-off for determining the neutralization endpoint titre of diluted serum samples was set to ≥50% neutralization (Fig. S8F), the cut-off of positivity for neutralization on live virus was 1:40 [51]. During assay validation, five out of the 10 NIBSC standards (CS678 WHO protocol) were positive in the assay with mid and high levels of live neutralization, consistent with the confirmed results from the NIBSC.”
- Figure S8: we have added this new supplementary figure.
- Page 3, line 51 (Supplementary Material): we have added Figure S8 legend. We have cited this new Figure in the Material and Methods (please see above).

2. **The authors make emphasis on the sensitivity and adequacy of the flow cytometry technique to assess quantification of immunoglobulins. The method is a cumbersome technique that requires transfection of HEK293 cells (it is unclear if is a stable or transitory transfection), cell permeabilization and staining of the cells suspension to be read in a flow cytometer. It is difficult to see how this method would be a viable tool to replace an indirect ELISA utilizing purified recombinant viral spike variants in terms of labor, cost, and sensitivity.**

We agree with Reviewer 2 that high capacity ELISAs detecting antibodies to Spike are practical, inexpensive, and automated. However, our work clearly shows that the quantitative flow cytometry assay is more sensitive for Spike antibody detection compared to several commercial immunoassays (Table 2). In addition, the flow cytometry assay is used for the detection of other neuroimmunological antibodies such as N-Methyl D-Aspartate Receptor-, aquaporin-4- and myelin oligodendrocyte glycoprotein (MOG)-antibodies for clinical diagnostic purposes (Waters P et al 2016, Reindl M et al 2020, Graus et al 2016, Tea F et al 2019, Jarius S et al 2018). In these cases, due to the lack of antigenic conformation, ELISAs have been recommended NOT to be used in routine diagnostic testing (Reindl et al 2020).

In our current study, transfection in HEK293 cells was transient. Our work shows that Spike variants can be easily included in our assay and that the data is correlated to fusion inhibition and live virus neutralization. While we have not compared Spike variant data between flow cytometry and commercial ELISAs (due to their unavailability), we still propose that our assay remains a viable diagnostic tool to complement data from immunassays, especially in the assessment of IgG binding to variants of concern (VOC). Indeed, we have received funding from the Australian State government to accredit and implement the assay in routine diagnostic testing (as we have just completed for the MOG antibody assay for diagnosis of MOG antibody-associated disease or MOGAD).

To take into consideration the points raised by Reviewer 2, we have edited the manuscript:

- Page 16, Line 373 (Discussion): we have added “despite their comparatively higher costs“ in the sentence “In this context, and future seroprevalence studies, more sensitive antibody assays will be essential despite their comparatively higher costs.”
- Page 17, Line 382 (Discussion): we have added the sentence “More so, due to its modular capacity in assessing Spike IgG binding to emerging variants, the assay could be useful during vaccine studies.”
- Page 23, Line 530 (Materials and Methods): we have clarified the text and added the word “transiently” in the sentence “SARS-CoV-2 full-length Spike (Wuhan-1 D614, V367F, G476S, V483A, D614G, S477N/D614G, S477N/D614G/V1068F, B.1.1.7 (United Kingdom), and B.1.351 (South Africa)) (10, 11), Membrane, and Envelope proteins were transiently expressed on transfected HEK293 cells.”

**Reviewer 3**

1. **The authors determine SARS-CoV-2 antibodies against surface viral proteins (S, M and E) using a flow-cytometry assay. As the authors propose that this test should be used for assessment of antibody responses at it enhances the sensitivity for detection of Spike antibodies (Discussion lines 353-356) in comparison with conventional ELISA tests (Table 2, Euroimmun test and Diasorin) standard controls should be incorporated to rule out false positive results in the flow cytometry assay.**

We thank Reviewer 3 for this comment. In our study, all the ADAPT and LIFE samples were RT-PCR-positive. To validate our assay according to international standards, we have tested 10 antibody preparations from the National Institute for Biological Standards and Control (NIBSC) that were distributed during the CS678 protocol for the WHO collaborative study to establish the first reference panel and international standard for anti-SARS-CoV-2 antibody. Of the 10 NIBSC standards, 8 were seropositive in the flow cytometry assay, one was a true negative and another one was low in Spike antibody, these results were concurrent with results confirmed by WHO. We have also tested 24 pre-pandemic adult sera (used to establish the positive threshold in Figure 1 and shown in Fig. S4C), 24 pediatric pre-pandemic sera, and 8 RT-PCR-negative adults collected during the pandemic, among which one patient was infected by coronavirus 229E. All pre-pandemic and PCR-negative sera were seronegative for Spike IgG.

To take into consideration the points raised by Reviewer 3, we have edited the manuscript:

- Page 23, line 539 (Materials and Methods): We have added, “Furthermore, 24 pediatric pre-pandemic controls and 8 PCR-negative pandemic adults, among which one patient was infected by coronavirus 229E, were also tested, and were all seronegative for Spike IgG. The assay was verified using 10 antibody standards from the National Institute for Biological Standards and Control (NIBSC). These were tested using the standard protocol for this study. These NIBSC standards were distributed during the CS678 protocol for the World Health Organization (WHO) collaborative study to establish the first International Standard for anti-SARS-CoV-2 antibody and Reference Panel, which our laboratory participated in. Of the 10 NIBSC standards, eight were positive in the assay either with low, mid, and high levels of Spike IgG, consistent with the confirmed results from the NIBSC.”

2. **From figure 1A and methods (lines 477-483) it is not clear if Membrane and/or Envelope proteins were expressed individually in different cell lines or in the same cell line. Please clarify.**

Reviewer 3 is correct. All antigens, i.e. Membrane, Envelope, and Spike, were expressed individually in different cell lines (by transient transfection, please see Reviewer 2, Pt 2). Therefore cells used in the experiments expressed Spike-only, or Membrane-only, or Envelope-only.

We have clarified the text:

- Page 23, line 526 (Materials and Methods): “Individual SARS-CoV-2 full-length Spike (Wuhan-1 D614, V367F, G476S, V483A, D614G, S477N/D614G, S477N/D614G/V1068F, B.1.1.7 (United Kingdom), and B.1.351 (South Africa)) [10, 11], Membrane, and Envelope proteins were transiently expressed on transfected HEK293 cells.”

3. **Related with the last point, in figure 1D membrane IgG antibodies are quantified. Did the authors quantify anti-Envelope antibodies as part of polyantigenic breadth? (lines 155-167).**

Only two ADAPT patients were positive for Envelope IgG, both patients were also positive for all other antigens and therefore, as Reviewer 3 mentioned, had polyantigenic responses.

We have amended the text and Figure 3A.

- Page 10, line 224 (Results): We have added a the sentence “Interestingly, the two individuals positive for Envelope IgG also had antibodies against all other SARS-CoV-2 proteins.”
- Page 39 line 913 (Figure legends): We have added an asterisk in Figure 3A (above the number 55 in the teal section of the ADAPT pie chart, and have edited Figure 3 legend “Approximately half of patients (49-55%) had broad SARS-CoV-2 antibodies (blue, * includes n = 2 patient seropositive for Envelope IgG).”

4. **Emerging variants studies are interesting but their relevance is low taking into account the current circulating Variants of Concern….To be published in Plos Medicine, to include the reactivity and neutralization capacity of the studied plasma against current variants of concern (B.1.1.7, B1.351 and B.1.1.248) would increase the interest of the article.**

We thank Reviewer 3 for these comments and we agree. In our submitted manuscript, we have assessed the binding of antibodies to several emerging and circulating Spike variants at that time. This included the now ubiquitous D614G, and also D614G/S477N, a variant prevalent in Australia during the second wave in August 2020. In 2020, several SARS-CoV-2 variants of concern (VOC) appeared primarily in the UK (B.1.1.7), South Africa (B1.351), and Brazil (B1.1.28.1 and B1.1.28.2), and the B.1.1.7 variant is currently becoming the most dominant worldwide. Although these variants were not transmitted in the community in Australia, they were detected in individuals infected overseas within the quarantine system. To address these VOCs, we have now tested the sera shown in the previous Figure 5H (n = 48, first and second waves) on our antibody assay including the B.1.1.7 and B.1.351 Spike variants and both virus-cell and live neutralization assays. Importantly, we also have functional neutralization data with live VOC B1.1.28.1 and B1.1.28.2 in the same cohort (n=48). Importantly, data on neutralization of the authentic VOC B1.1.28.1 and B1.1.28.2 have been seldom reported before.

Overall, the data shows that the two groups of patients displayed a decrease binding to Spike, reduced fusion inhibition and neutralization toward the VOC B.1.1.7 and B.1.351, but more so for B.1.351, and also a decrease of neutralization of VOC B1.1.28.1 and B1.1.28.2. This suggests a global decrease of immunoreactivity toward VOC as observed for the D614G/S477N Spike. Interestingly, we have tested a small group of high and elite responders as defined in Figure 4, and showed that their capacity to neutralize the live VOC is not as impaired as the other individuals, suggesting once again that there is a small group of high responders with an exquisite response to SARS-CoV-2. We have compiled this results in an updated Figure 5.

As Reviewer 3 points out, this new data is important and very topical as several vaccines have shown reduced protection against these variants, which is supported by our data. Also reporting on convalescent responses in Australia, a country with a very low rate of infection and re-infection, will inform future studies focusing on breadth and longevity of vaccine responses.

We have edited the manuscript to reflect these changes:

- Page 4, line 67 (Abstract), we have now written: “The SARS-CoV-2 antibody neutralization response and its evasion by emerging viral variants and variant of concern (VOC) are unknown, but critical to understand re-infection risk and breakthrough infection following vaccination.” and on line 77, we have added “and VOC.”
- Page 5, line 103 (Introduction): we have have added: “One example is the S477N/D614G Spike variant which appeared in Australia during July and August 2020, was traced to a single event from Australian hotel quarantine [12], and represented greater than 58% in Oceania [13]. More recently, several SARS-CoV-2 variants of concern (VOC) appeared primarily in the UK (B.1.1.7), South Africa (B.1.351), and Brazil (B1.1.28.1 and B1.1.28.2), and VOC B.1.1.7 is currently becoming the most dominant worldwide [14].”
- We have edited Fig. 1A to include new variants (page 34) and have edited its legend (page 35 from line 885).
- We have added several new panels to Fig 5, from panel E to L (page 42) and have edited its legend (page 43 from lines 946 to 956).
- We have edited data Fig S4 to include new variants, have edited its legend (Page 2, line 26, Supplementary Material), and updated the citation in the main text (Page 12, line 272; Page 13, line 302; Page 14, line 325).
- Page 12, line 267 (Results), we have added: “and recent emerging variants from the United Kingdom, South Africa, and Brazil [14].”
- Page 14 (Results), we have added results from the live neutralization experiments with the S477N/D614G variant on from line 312 to 319).
- Page 14-15 (Results), we have added a result section on (from lines 321 to 334): “In 2020, B.1.1.7, B.1.351, and B1.1.28.1 and B1.1.28.2 appeared at three geographical locations. These VOC only represented 7% community transmission in Australia, whereas quarantine detection accounted for 93% in March 2021 (Fig. 5E). All ADAPT patients from the first (n =31) and second wave (n=17) were seropositive and had detectable IgG against Spike VOC B.1.1.7 and B.1.351 (Fig. S4D). Compared to the D614G variant (parental variant), a strong decrease in immunoreactivity to VOC B.1.1.7 and B.1.351 was observed in all ADAPT patients (Fig. 5I). Most patients had reduced Spike virus-cell fusion inhibition and neutralization toward VOC B.1.1.7 and B.1.351, but the proportion of reduced patients and the level of the decrease was greater against VOC B.1.351 (83%, 39/47, and 96%, 45/47, respectively (Fig. 5J and 5K). There was also a strong decrease of neutralization to both live VOC B.1.1.28.1 and B.1.1.28.2 (Fig. 5L). Interestingly, four elite responders were able to maintain neutralization against the four live VOC viruses (Fig. S7), suggesting that only a small proportion community infected individuals would harbor immune protection in the event of a re-infection with emerging VOC.”
- Page 14-15 (Results), we have edited the citations to Fig 5 in the Results section (from lines 296 to 334).
- Page 18, line 415 (Discussion): “Although the pseudotyping fusion assay had lower sensitivity, and indeed was observed with all variants tested, most individuals across both cohorts and validation controls had titers in this assay with potency ranking similar to full virus neutralization.”
- Page 19, line 448 (Discussion), we have added “The initial SARS-CoV-2 fitness gain of D614G appeared very early in the pandemic [9, 10], and serves as the foundation of all current viral variants, including VOC.”
- Page 20-21, from line 460 to line 490 (Discussion), we have now written: “Given the emergence of the S477N/D614G polymorphism in most patients infected in the Australian second wave and in Europe [12, 13], these results highlight concerns regarding fitness gain and immune evasion. Seroconversion was observed in all patients from the first and second wave, and good antibody binding to Wuhan-1 D614 and D614G, but there also was a significant decrease in binding, fusion inhibition, and neutralization to S477N/D614G Spike independent of the variant that had infected individuals. Whilst our data on S477N/D614G emphasize a singular polymorphism effect on fitness gain and immune evasion, the eight and more changes in Spike both within and outside the RBD, but without S477N, in the most recent VOC B.1.1.7, B.1.351, and B1.1.28.1 underlie that many changes could also result in significant observations of immune evasion, via less antibody immunoreactivity and a resistance to virus neutralization as shown by our data. The exact polymorphism(s) behind these observations is unknown. Across all VOCs, N501Y is a shared fitness gain that does not contribute to immune evasion [40], whereas E484K in VOC B.1.351, B1.1.28.1, and B1.1.28.2., as well as S477N, is associated with the ability to evade monoclonal antibodies [11, 41]. Additionally, the appearance of a N-glycosylation site within Spike RBD at position 477N could lead to glycan shielding, as in HIV [42]. This is not consistent with our data. A more likely mechanism would be a conformational change that not only leads to an increase affinity for ACE2 binding, but also enables Spike to render the virus broadly resistant to numerous antibodies [43]. Such polymorphisms are of signficant concern, as they increase viral fitness and also raise the probability of re-infection in convalescent and vaccinated individuals. Unfortunately re-infections are currently prevalent in Brazil [44], and the results from vaccination efficacy trials in South African both support real-world observations of the challenges we face when variants like B1.1.28.1, B1.1.28.2. and B.1.351 enter and spread within communities. Our findings readily imply a need for periodic updates in vaccine design, as for the influenza vaccine [45]. It is highly probable that we will need to address future VOC that provide even greater challenges for current vaccine design; additional spike changes, such as Q498R and/or the merging of S477N with E484K and N501Y, that can lead to further fitness gains. For instance a Spike RBD with S477N, E484K, Q498K, and N501Y produces a SARS RBD that is 600-fold greater at engaging ACE2 [41, 43]. In that context, how such a SARS- CoV-2 infection would proceed and whether current vaccines would provide sufficient protection is presently unknown.”
- Page 21, line 498 (Discussion, we have edited the text and have now written: “and re-infection risk and breakthrough infection following vaccination.”
- Page 23, line 530 (Material and Methods): “…**,** B.1.1.7 (United Kingdom), and B.1.351 (South Africa))**”**
- Page 24, line 563 (Materials and Methods), we have added: “including D614, D614G, S477N/D614G, B.1.1.7, and B.1.351 [47].”

5. **First sample was obtained beyond two months after a positive PCR test. Thus, one limitation of the study is the assessment of early antibody responses anti-SARS-CoV-2.**

We have now mentioned this limitation in the manuscript and have edited the manuscript:

- Page 16, line 355 (Discussion): “Early antibody responses against SARS-CoV-2 could not be studied as the first sample was collected two months after PCR positivity, and is a limitation of the study.”

6. **Relationship between higher immunoglobulin levels and age probably is due to more severe disease in elderly people. This point should be mentioned.**

We agree. There was a relationship between higher immunoglobulins levels and age. We had mentioned that the high responders were more likely to be male, hospitalized, and were of older age, and had cited Fig. 4A (Page 11, line 251). We have also edited the title of the section (Page 11, line 246) and have now written: “High responders with strong and broad SARS-CoV-2 antibody responses are rare and could be ideal plasma donors.”

7. **Articles that characterize the presence of memory B-lymphocytes targeting the Spike protein should be mentioned as a proof of long-lasting immune memory (Papers from Shane Crotty for example).**

Thank you. We have now added the reference by J Dan (S Crotty and A Sette).

- Page 19 line 438 (Discussion): we have added the reference by J Dan et al, 2021 and we have cited it as the new reference 37.

**Other edits**

We have nuanced the use of convalescent plasma for COVID-19 therapy in the discussion and have updated the references.

- Page 18, line 419: we have now written: “Whilst initial clinical trials have supported an acceptable safety profile, there is increasing evidence of a lack of therapeutic efficacy in hospitalised patients. One systematic review and meta-analysis, and the Randomized Evaluation of COVID-19 Therapy (RECOVERY) trial, the largest randomised, controlled trial involving convalescent plasma to date recently reported no significant difference in 28-day mortality between hospitalized patients receiving high-titer convalescent plasma and those receiving standard care [28, 29]. There remains a question whether high titer units given early or COVID-19 IgG could still provide a benefit for preventing disease progression. Some successful trials used high-titer of convalescent COVID-19 plasma delivered within three days of hospitalization and showed reduction of disease progression [30-33]. Alternatively, there may be a role in immunosuppressed patients who cannot produce their own antibody response, in particular for patients with Agammaglobulinemia [34, 35]. Our findings that the immunological response to SARS-CoV-2 is widely heterogeneous, with large variations in SARS-CoV-2 antibodies and neutralization, polyantigenic immunoreactivity, and longitudinal responses complement these assertions. To take into account the first phase of decay observed during early convalescence, we propose an optimal window for plasmapheresis, up to 100 days post-diagnosis. Furthermore, the occurrence of a small group of individuals, termed “high and elite responders” with highly and broad neutralising, broad, and sustained SARS-CoV-2 antibody responses over time, may be due to the rapid and lasting generation of memory B cells [36, 37]. These patients were likely to be hospitalized older males. Whilst vaccination and in particular boosting of naturally infected invididuals offers a potential alternative source of convalescent plasma, should convalescent plasma be demonstrated to have a role in treatment, these “high and elite” responders could provide a valuable source of convalescent plasma in the future.”
