# Supplementary material for: SARS-CoV-2 neutralizing antibodies: Longevity, breadth, and evasion by emerging viral variants
Source: PLoS Med. 2021 Jul 6;18(7):e1003656. doi: 10.1371/journal.pmed.1003656 (PMC8291755; doi:10.1371/journal.pmed.1003656)
Supplement: Attachment — Submitted filename: FTea SARS-COV2_PlosMedicine response to AE_RevII.docx [file pmed.1003656.s012.docx]

**Point-by-point response to the Associate Editor:**

We would like to thank the Associate Editor for re-reviewing our manuscript. All amendments to the manuscript have been underlined (please note that the page number refers to the revised version of our manuscript in the marked up manuscript file).

**Associate Editor points**

1. **There needs to be consistent citation of p values where they state a difference is significant. There are several places where they report significant differences, but no p value provided in the text, and often it is missing in the figure/legend. They also describe higher or lower antibodies in different contexts in the text of the paper without indicating whether the differences are significant.**

We thank the Associate Editor for raising these comments. We have improved the consistency of the use of P values. We now cite the P values in the figure legends. As suggested by the Associate Editor (please see Point 2 below), we have elected to cite the P values in the figure legends rather than in the main text or on the figure themselves. We also have removed the term “significant or significantly” from statements which did not describe a statistical significance.

We have now edited the manuscript:

- Page 28, line 641 (Material and Methods): we have clarified the text in the statiscal analyses and have now written: “Dwass-Steel-Critchlow-Fligner and Mann-Whittney tests were used to test for significance between continuous and categorical variables.”
- Page 36, line 907 (Fig 2 legend), we have now added: “Neutralization titers were higher than viral-cell fusion titers in ADAPT (ns, not significant) and LIFE (P<0.0001)”.
- Page 37, line 916 (Fig 2 legend): we have now added “R2 values are shown and **** indicates significance (P<0.0001).”
- Page 43, lines 955-973 (Fig 5 legend): we have added P values for the comparisons between emerging variants and VOCs.
- Page 16, line 377 (Discussion): we have removed the word “significantly”.
- Page 20, line 452 (Discussion): we have removed the word “significantly”.
- Page 20, line 469 (Discussion): we have removed the word “significant”.

2. **They need to either indicate the p value or significance (or NS) in the figures themselves, or include them in the figure legend. Since many of their figures are very small, adding p values to the figure legend would be better and clearer.**

We have now added the P values and the non significance (ns) in the figures legends as suggested by the Associate Editor. Please see Points 1 and4 above for exact changes to the text of the figure legends.

3. **Many figures are lacking any indication of a p value, either in the figure, the legend, or in the manuscript text. My recommendation to make this more consistent and accessible to readers is for them to put p values for each figure in the legend. this needs to be done for all figure sub-parts. If the difference is not significant, they should state the exact p value**

We have now added the P values and the non significance (ns) in the figures legends as suggested by the Associate Editor. Please see Points 1 and4 above for exact changes to the text of the figure legends.

4. **When comparing differences in antibodies between clinical groups, there is no mention if they adjusted for covariates, or how they did this.**

Thank you for this comment. The comparison in antibody differences between clinical groups have been performed using the Dwass-Steel-Critchlow-Fligner test for pair-wise comparisons (Fig 3). Co-variates were not included in that analysis. To answer the Associate Editor’s comment, we have now performed an ANOVA and have fitted a linear model using the co-variates (eg. age and gender), and the MFI or virus-cell fusion inhibition/neutralization titers as response variables. Some of the variance was due to age and gender, and some P values now are not significant. We have adjusted the P values in Figure 3C and D and legend. Please note that we have done these analyses for the ADAPT cohort, but not for the LIFE cohort as the sample size of LIFE hospitalized donors was too low to achieve statistical power.

We have edited the text:

- Page 28, line 642 (Material and Methods): we have added “Comparisons between clinical groups (Fig. 3) were performed using general Linear Models (GLMs) (gaussian) to model the response (MFI) and explanatory variables, such as sex and age. ANOVA likelihood-ratio test was used to test whether interactions need be included in the final model. Adjusted P values were calculated using Tukey’s HSD test.”
- Page 38 (Fig 3): we have uploaded an updated fig 3 in which we have edited the P values on panels C and E.
- Page 39, line 932 (Fig 3 legend): we have added “). *: P<0.05, **: P<0.01, ***: P<0.001, ****: P<0.0001, *****: P<0.00001. Not significant if significance is not indicated. P values in LIFE hospitalized patients (C) were not calculated due to low sample size.”
